# HDCytoData: Collection of high-dimensional cytometry benchmark datasets in Bioconductor object formats

**DOI:** 10.12688/f1000research.20210.2

**Published:** 2019-12-04

**Authors:** Lukas M. Weber, Charlotte Soneson

**Affiliations:** 1 Institute of Molecular Life Sciences, University of Zurich, Zurich, 8057, Switzerland; 2 SIB Swiss Institute of Bioinformatics, Zurich, 8057, Switzerland; 3 Friedrich Miescher Institute for Biomedical Research, Basel, 4058, Switzerland; 4 SIB Swiss Institute of Bioinformatics, Basel, 4058, Switzerland

**Keywords:** benchmarking, high-dimensional cytometry, Bioconductor, ExperimentHub, clustering, differential analyses

## Abstract

Benchmarking is a crucial step during computational analysis and method development. Recently, a number of new methods have been developed for analyzing high-dimensional cytometry data. However, it can be difficult for analysts and developers to find and access well-characterized benchmark datasets. Here, we present HDCytoData, a Bioconductor package providing streamlined access to several publicly available high-dimensional cytometry benchmark datasets. The package is designed to be extensible, allowing new datasets to be contributed by ourselves or other researchers in the future. Currently, the package includes a set of experimental and semi-simulated datasets, which have been used in our previous work to evaluate methods for clustering and differential analyses. Datasets are formatted into standard SummarizedExperiment and flowSet Bioconductor object formats, which include complete metadata within the objects. Access is provided through Bioconductor's ExperimentHub interface. The package is freely available from http://bioconductor.org/packages/HDCytoData.

## Introduction

Benchmarking analyses are frequently used to evaluate and compare the performance of computational methods, for example by users interested in selecting a suitable method, or by developers to demonstrate performance improvements of a newly developed method. A critical part of any benchmark is the selection of appropriate benchmark datasets ^1, 2^. In some cases, suitable publicly available datasets may be found in the literature. Alternatively, new experimental or simulated datasets containing a known ground truth may be created by the authors of the benchmark ^1, 2^.

High-dimensional cytometry refers to a set of recently developed technologies that enable measurement of expression levels of up to dozens of proteins in hundreds to thousands of cells per second, using targeted antibodies labeled with various types of reporter tags. This includes multi-color flow cytometry, mass cytometry (or CyTOF), and sequence-based cytometry (or genomic cytometry). Due to the large size and high dimensionality of the resulting data, numerous computational methods have been developed for analyzing these datasets ^3^. Many of these methods are based on the fundamental concept of analyzing cells in terms of cell populations, for example using clustering to define cell populations, or detecting differential cell populations between conditions.

In our previous work, we have collected a number of benchmark datasets to evaluate methods for clustering ^4^ and differential analyses ^5^ in high-dimensional cytometry data. This includes publicly available datasets previously published by other groups or our experimental collaborators, as well as new semi-simulated datasets that we generated. In these previous publications, we recorded links to original data sources and made all data available via FlowRepository ^6^. FlowRepository is a widely used resource in the cytometry community, which provides a permanent record of publicly available datasets associated with peer-reviewed publications, and which has also been used by other authors to distribute benchmark datasets (e.g., 7, 8). However, FlowRepository is primarily accessed via a web interface, and downloading and loading data for further analysis in R requires customized code and matching of metadata (e.g., sample information), which can hinder accessibility and reproducibility.

Here, we introduce the HDCytoData package, which provides a resource for re-distributing high-dimensional cytometry benchmark datasets through Bioconductor’s ExperimentHub ^9^, in order to improve accessibility. ExperimentHub provides a flexible platform for hosting datasets in the form of R/Bioconductor objects, which can be directly loaded within an R session. We have formatted the datasets in HDCytoData into standard SummarizedExperiment and flowSet Bioconductor object formats ^10– 12^, which include all required metadata within the objects and facilitate interoperability with R/Bioconductor-based workflows. The data objects are intended to be static, with no major updates following release. We envisage that these datasets will be useful for future benchmarking studies, as well as other activities such as teaching, examples, and tutorials. The package is extensible, allowing new datasets to be contributed by ourselves or other researchers in the future. It is designed to be accessible for users who are familiar with R and Bioconductor, but who may not have used ExperimentHub packages before. The package is freely available from http://bioconductor.org/packages/HDCytoData.

## Methods

### Implementation

The benchmark datasets currently included in the HDCytoData package consist of experimental and semi-simulated data, and can be grouped into datasets useful for benchmarking algorithms for (i) clustering and (ii) differential analyses. Table 1 and Table 2 provide an overview of the datasets.

**Table 1.** Summary of benchmark datasets for evaluating clustering algorithms. For more details on these datasets, see Table 2 in 4, or the HDCytoData help files.

Dataset	ExperimentHub ID	Number of cells	Number of dimensions	Number of reference cell populations	Type of ground truth	FlowRepository ID	Original reference
Levine_ 32dim	EH2240 – EH2241	265,627	32	14	Manual gating	FR-FCM-ZZPH	13
Levine_ 13dim	EH2242 – EH2243	167,044	13	24	Manual gating	FR-FCM-ZZPH	13
Samusik_ 01	EH2244 – EH2245	86,864	39	24	Manual gating	FR-FCM-ZZPH	14
Samusik_ all	EH2246 – EH2247	841,644	39	24	Manual gating	FR-FCM-ZZPH	14
Nilsson_ rare	EH2248 – EH2249	44,140	13	1 (rare population)	Manual gating	FR-FCM-ZZPH	15
Mosmann_ rare	EH2250 – EH2251	396,460	14	1 (rare population)	Manual gating	FR-FCM-ZZPH	16

**Table 2.** Summary of benchmark datasets for evaluating methods for differential analyses. For more details on these datasets, see Supplementary Note 1 in 5, or the HDCytoData help files.

Dataset	ExperimentHub ID	Type of data	Number of cells	Number of dimensions	Type of ground truth	Type of differential analysis	FlowRepository ID	Original reference
Krieg_Anti_ PD_1	EH2252 – EH2253	Experimental	85,715	24 (cell type)	Qualitative	Differential abundance	FR-FCM-ZYL8	17
Bodenmiller_ BCR_XL	EH2254 – EH2255	Experimental	172,791	24 (10 cell type; 14 cell state)	Qualitative	Differential states	FR-FCM-ZYL8	18
Weber_AML_ sim	EH3025 – EH3046	Semi- simulated (multiple simulation scenarios)	157,593 (excluding spike-in)	16 (cell type)	Spike-in cell labels	Differential abundance	FR-FCM-ZYL8	5
Weber_BCR_ XL_sim	EH3047 – EH3064	Semi- simulated (multiple simulation scenarios)	85,331 (main simulation; excluding spike-in)	24 (10 cell type; 14 cell state)	Spike-in cell labels	Differential states	FR-FCM-ZYL8	5

The raw datasets were collected from various sources ( Table 1 and Table 2), and have been extensively reformatted and documented for inclusion in the HDCytoData package. Each dataset is stored in both SummarizedExperiment and flowSet formats, since these are the most commonly used R/Bioconductor data structures for high-dimensional cytometry data (and there is generally no straightforward way to convert between the two). The objects each contain one or more tables of expression values, as well as all required metadata. Following standard conventions used for cytometry data ^19^, rows contain cells, and columns contain protein markers. Row metadata includes sample IDs, group IDs, patient IDs, reference cell population labels (where available), and labels identifying ‘spiked in’ cells (where available). Column metadata includes channel names, protein marker names, and protein marker classes (cell type, cell state, as well as non protein marker columns). Note that raw expression values should be transformed prior to performing any downstream analyses. Standard transformations include the inverse hyperbolic sine ( asinh) with cofactor parameter equal to 5 for mass cytometry or 150 for flow cytometry data ( 20, Supplementary Figure S2); several other alternatives also exist ^21^.

Most of these datasets include a known ground truth, enabling the calculation of statistical performance metrics. The ground truth information consists of reference cell population labels for the clustering datasets, and labels identifying computationally ‘spiked in’ cells for the differential analysis datasets. The datasets without a ground truth instead consist of experimental datasets that contain a known biological signal, which can be used to evaluate methods in qualitative terms; i.e., whether methods can reproduce the known biological result.

Extensive documentation is available via the help files for each dataset—including descriptions of the datasets, details on accessor functions required to access the expression tables and metadata, and links to original sources. In addition, reproducible R scripts demonstrating how the formatted SummarizedExperiment and flowSet objects were generated from the original raw data files from FlowRepository are included within the source code of the package.

New datasets may be contributed by ourselves or other authors in the future. The procedure for external contributions is described in the vignette titled “Contribution guidelines”, available from Bioconductor. This vignette describes the submission procedure (via GitHub), as well as the required files (data objects in SummarizedExperiment and flowSet formats containing all necessary metadata, reproducible R scripts showing how the formatted objects were generated from the original raw data files, documentation, and package metadata).

### Operation

The HDCytoData package can be installed by following standard Bioconductor package installation procedures. All datasets listed in Table 1 and Table 2 are available in Bioconductor version 3.10 and above. Minimum system requirements include a recent version of R (3.6 or later; this paper was prepared using R version 3.6.1), on a Mac, Windows, or Linux system. Example installation code is shown below.


# install BiocManager              
install.packages("BiocManager")    

# install HDCytoData package       
BiocManager::install("HDCytoData") 


Once the HDCytoData package is installed, the datasets can be downloaded from ExperimentHub and loaded directly into an R session using only a few lines of R code. This can be done by either (i) referring to named functions for each dataset, or (ii) creating an ExperimentHub instance and referring to the dataset IDs. Example code for each option for one of the datasets is shown below. Note that each dataset is available in both SummarizedExperiment and flowSet formats. After an object has been downloaded, the ExperimentHub client stores it in a local cache for faster retrieval. File sizes for these datasets range from 2.4 MB ( Nilsson_rare) to 194.5 MB ( Samusik_all) (see help files). The local download cache can be cleared using the removeCache function from the ExperimentHub package (see HDCytoData package help file or main vignette). For more details on accessing ExperimentHub resources, refer to the ExperimentHub vignette available from Bioconductor.


# load HDCytoData package                                   
library(HDCytoData)                                         

# option 1: load datasets using named functions             
d_SE <- Bodenmiller_BCR_XL_SE()                             
d_flowSet <- Bodenmiller_BCR_XL_flowSet()                   

# option 2: load datasets by creating ExperimentHub instance
ehub <- ExperimentHub()                                     
query(ehub, "HDCytoData")                                   
d_SE <- ehub[["EH2254"]]                                    
d_flowSet <- ehub[["EH2255"]]                               


Once the datasets have been downloaded and loaded, they are available to the user as R objects within the R session. They can then be inspected and manipulated using standard accessor and subsetting functions (for either the SummarizedExperiment or flowSet object class). Example code to inspect a SummarizedExperiment is displayed below. For more details on how to load and inspect datasets, including the expected output from each function shown here, refer to the HDCytoData package main vignette available from Bioconductor.


# inspect SummarizedExperiment object
d_SE                                 
assays(d_SE)                         
rowData(d_SE)                        
colData(d_SE)                        
metadata(d_SE)                       


Documentation describing each dataset is available in the help files for the objects, which can be accessed using the standard R help interface, as shown below.


# display documentation (help files)
?Bodenmiller_BCR_XL                 
help(Bodenmiller_BCR_XL)            


## Use cases

The datasets currently included in the HDCytoData package ( Table 1 and Table 2) can be used to benchmark methods for either (i) clustering or (ii) differential analyses. In addition, these datasets may be useful for other activities such as teaching, examples, and tutorials (e.g., demonstrating how to use a new computational tool).

For the clustering benchmark datasets ( Table 1), performance can be evaluated by calculating metrics such as the mean F1 score or adjusted Rand index, which measure the similarity between two sets of cell labels (i.e., the cluster labels and the ground truth or reference cell population labels) ^1^. A short example is shown in the vignette titled “Examples and use cases”, available from Bioconductor. For more extensive examples and evaluations, see the GitHub repository accompanying our previous study ^4^.

**Figure 1.**
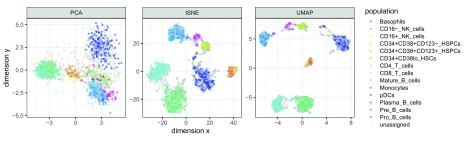
Example of use case for datasets in the HDCytoData package. This example compares three different dimension reduction algorithms — principal component analysis (PCA), t-distributed stochastic neighbor embedding (tSNE), and uniform manifold approximation and projection (UMAP) — for visualizing cell populations in the Levine_32dim dataset ( Table 1). Colors indicate the known ground truth cell populations.

These datasets can also be used to generate visualizations demonstrating the performance of dimension reduction algorithms. For example, Figure 1 compares three different dimension reduction algorithms (principal component analysis [PCA], t-distributed stochastic neighbor embedding [tSNE] ^22, 23^, and uniform manifold approximation and projection [UMAP] ^24, 25^), for one of the datasets ( Levine_32dim), with colors indicating the ground truth cell population labels. The figure shows a clear visual separation between the populations, with varying performance for the different algorithms. Reproducible R code for this figure is available in the “Examples and use cases” vignette, and the GitHub repository http://github.com/lmweber/HDCytoData-example.

For the differential analysis benchmark datasets ( Table 2), methods can be evaluated by their ability to recover the known differential signals, either in quantitative terms using the ground truth spike-in cell labels (for the semi-simulated datasets), or in qualitative terms (for the experimental datasets). The differential signals consist of either differential abundance of cell populations, or differential states within cell populations (i.e., differential expression of additional functional markers within cell populations), providing conceptually distinct differential analysis tasks. A short example showing how to perform differential analyses on these datasets is provided in the “Examples and use cases” vignette. For more extensive examples and evaluations, see the GitHub repository accompanying our previous study ^5^.

## Summary

The HDCytoData package is an extensible resource providing streamlined access to a number of publicly available benchmark datasets used in our previous work on high-dimensional cytometry data analysis. Datasets are provided in standard Bioconductor object formats, and are hosted on Bioconductor’s ExperimentHub platform. In the future, it may make sense to develop similar packages for other data types, e.g., imaging mass cytometry, once several well-characterized benchmark datasets become available. By facilitating access to these datasets, we hope they will be useful for other researchers interested in designing rigorous benchmarks for method development or other computational analyses, as well as other activities such as teaching, examples, and tutorials.

## Data availability

All data underlying the results are available as part of the article and no additional source data are required.

## Software availability

**Software available from**: http://bioconductor.org/packages/HDCytoData

**Source code available from**: https://github.com/lmweber/HDCytoData

**Archived source code at time of publication**: https://doi.org/10.5281/zenodo.3551051 ^26^

**Licence**: MIT License
