# Supplementary material for: HDCytoData: Collection of high-dimensional cytometry benchmark datasets in Bioconductor object formats
Source: F1000Res. 2019 Dec 4;8:1459. Originally published 2019 Aug 19. [Version 2] doi: 10.12688/f1000research.20210.2 (PMC6904983; doi:10.12688/f1000research.20210.2)
Supplement: Peer review history

# Peer review: Reviewer response for version 2

**DOI:** 10.5256/f1000research.23700.r57458

**Published:** 2019-12-10

**Authors:** Shila Ghazanfar

**Affiliations:** 1 Cancer Research UK Cambridge Institute (CRUK CI), Cambridge, UK

The authors have done an excellent job in addressing my comments and concerns. The manuscript contains further detail, including information suitable to those less familiar with high dimensional cytometry data, and two new vignettes have been added to the HDCytoData package containing further examples and use-cases, as well as guidelines towards contribution.

I confirm that I have read this submission and believe that I have an appropriate level of expertise to confirm that it is of an acceptable scientific standard.

# Peer review: Reviewer response for version 2

**DOI:** 10.5256/f1000research.23700.r57457

**Published:** 2019-12-04

**Authors:** Laurent Gatto

**Affiliations:** 1 De Duve Institute, University of Louvain (UCLouvain), Brussels, Belgium

Thank you for the amendments.

 I hope the new vignette and guidelines will promote the contribution by other developers/users to further improve this, already very useful, package.

I confirm that I have read this submission and believe that I have an appropriate level of expertise to confirm that it is of an acceptable scientific standard.

# Peer review: Reviewer response for version 1

**DOI:** 10.5256/f1000research.22200.r52679

**Published:** 2019-09-02

**Authors:** Laurent Gatto

**Affiliations:** 1 De Duve Institute, University of Louvain (UCLouvain), Brussels, Belgium

Weber and Soneson present HDCytoData, a Bioconductor data package providing pre-formatted high-dimensional cytometry data. The preparation of the datasets as SummarizedExperiment and flowSet objects makes these amendable for benchmarking, a crucial step when developing new methods.

 My main comment centres around the contribution of new data. While the curated/formatted data in the package have already been useful to the authors in their previous work, the ambition is to make it possible for others to benefit from them and, to enable this in the longer term, to expand the package with additional data. These contributions are anticipated to come from the original authors and, ideally, also by new contributors.

 The contribution procedure, while crucial, (1) isn't described very clearly and, at least in its current form, (2) only applies to seasoned R users/programmers. These two points constitute a serious barrier to external contributions.

 Indeed, the only information that is provided are a list of three required artefacts (objects, scripts and documentation), without details as to how to produce these, nor how to provide them. I would suggest to add a 'How to contribute' vignette to the package, describing all these aspects, including an example for one of the existing data. I would also suggest to include a contribution code of conduct, given that external contributions are explicitly advertised.

 I would suggest asking new contributors to send a pull request (PR) on Github, with possible alternative methods for those that aren't familiar with GitHub. The use a PR provides traceability (as opposed to an email, for instance) and publicly recognises the external contribution, as PRs are publicly recorded on GitHub. I would also suggest to explicitly define how external contributions are to be acknowledged in the contribution guide (for example addition as a 'contributor' in the DESCRIPTION file).

 These additions will clarify what is expected for a contribution to be considered, how it will be managed by the authors, and how it will be acknowledged, thus hopefully facilitating the process.

 Minor suggestions:

- How can a potential user find out if/when new data have been added to the package? While `?HDCytoData` gives a list of dataset, a function returning a vector or dataframe with dataset names and possibly some annotation would be useful for programmatic access (given here that `data(package = "HDCytoData")` doesn't work for data on ExperimentHub).
- It could be useful to expand the 'Use cases' section with (1) example calculations of the F1 scores and Rand indices for the clustering example and (2) adding a similar short example for the differential analysis use case.
- I am curious as to why the content of the lmweber/HDCytoData-example isn't included as a vignette in the HDCytoData package (and thus lacking the usual control and documentation that comes with R packages).

I confirm that I have read this submission and believe that I have an appropriate level of expertise to confirm that it is of an acceptable scientific standard, however I have significant reservations, as outlined above.

# Response

**Published:** 2019-11-26

**Authors:** Lukas Weber

**Affiliations:** University of Zurich, Switzerland

Thank you for your comments and suggestions. As suggested, we have provided significant additional material on the procedure for contributing new datasets. We have expanded the section in the text on external contributions, and added an additional Bioconductor vignette titled “Contribution guidelines”. This vignette describes the required files (data objects, scripts, documentation, metadata), as well as the submission procedure. We have requested that contributions be submitted via GitHub issues and pull requests, clarified the acknowledgment procedure, and added a code of conduct.

Regarding the minor suggestions, we have also (i) updated the main vignette and package help file to show how to programmatically retrieve a data frame of all available datasets, and (ii) added a new vignette titled “Examples and use cases”, which includes the example from the previous repository ( https://github.com/lmweber/HDCytoData-example), as well as new examples showing how to use the datasets in the HDCytoData package to evaluate clustering performance (e.g. adjusted Rand index) and perform differential analyses.

# Peer review: Reviewer response for version 1

**DOI:** 10.5256/f1000research.22200.r52681

**Published:** 2019-08-28

**Authors:** Shila Ghazanfar

**Affiliations:** 1 Cancer Research UK Cambridge Institute (CRUK CI), Cambridge, UK

Weber and Soneson have written a software article presenting HDCytoData (currently version 1.4.0), a Bioconductor package aimed at making multiple high-dimensional cytometry (HDC) datasets available in a consistent R friendly format as either SummarizedExperiment or flowSet objects. The authors have framed this as an aid for facilitating benchmarking studies and for use for examples or tutorials in future. HDCytoData provides links to these datasets through ExperimentHub and includes some helpful commands for downloading such data. Currently eight datasets are included in the package which are accessed with easy-to-use function calls in the workspace.

 With this in mind, I have some comments & questions below that could improve the manuscript and useability of the HDCytoData package.

- This approach effectively duplicates the data from flowRepository and into the Bioconductor ExperimentHub ecosystem, is it more worthwhile to provide the functions to extract and process the data from the original flowRepository source? This manuscript could contain more motivation for hosting the processed data versus providing functions to download + process the data from flowRepository.
- Is it extensible to other additional characteristics? e.g. data arising from imaging mass cytometry with further measured features, or similar. The authors could discuss the breadth of experimental data types they imagine HDCytoData to encompass or accept from contributors. 
- The authors should discuss the continued curation of the data within the HDCytoData package and mention how it behaves in case of changes or updates to the 'original' data in flowRepository. Describe further how one can contribute their dataset(s)?
- I'm confused as to the framing of this package as principally for benchmarking studies. Whilst it's an important aspect of understanding and improving methodology, users of this package may be more interested in a convenient and consistent way of loading the flow cytometry data altogether, especially so for some integrative analysis of multiple HDC datasets.
- It's unclear how large the data files are that are being downloaded into the local cache, ideally the user would want to know this information before going ahead and downloading it.
- It's not clear in this manuscript how one would remove the data once it's no longer needed, or how to clear the cache. It appears that it's assumed users are also fairly familiar with the ExperimentHub interface. The authors should make it more clear what level of experience they imagine package users should have, i.e. who are they aiming the software towards?
- It would be useful to have a bulk download to cache, or possibly a bulk load to workspace option for these datasets.
- Is there a functionality to switch from SummarizedExperiment object to flowSort format? If this exists in another package it should be pointed to. 
- For the Bodenmiller data, I was surprised to find that the help file for Bodenmiller_BCR_XL_SE() says there are measurements for 24 proteins but the exprs assay has 35 columns, looking at the colData, features are classed into "type" and "state" with the remainder having a class of "none". Some of these columns do not appear to measure specifically protein abundance but rather cell-specific (as opposed to sample-specific) features, for instance "Cell_length". Have the authors anticipated this type of extra information and how it would fit into the SummarizedExperiment or flowSort object? The slot name "exprs" suggests that the data within this slot should be some molecular quantities, should these other features go into the rowData() slot instead? Or furthermore, whether ideally for downstream analysis (such as differential expression) these extra columns should be discarded? (Note these extra columns than what is listed in the function help descriptions appears for multiple datasets.)
- How do you ensure that there is enough information available here to be able to accurately normalise/standardise the data, especially so for flow cytometry data, given the particular combinations of fluorescent markers associated with the proteins, and potential overlap of the fluorescence for these markers?

I confirm that I have read this submission and believe that I have an appropriate level of expertise to confirm that it is of an acceptable scientific standard, however I have significant reservations, as outlined above.

# Response

**Published:** 2019-11-26

**Authors:** Lukas Weber

**Affiliations:** University of Zurich, Switzerland

Thank you for your comments and suggestions. We have updated the text, vignettes, and help files to clarify each of the issues raised above. Below are also responses to the specific questions:

(1) Code to process the raw .fcs files from FlowRepository into the SummarizedExperiment and flowSet formats is provided in the ‘make-data’ scripts saved in the ‘inst/scripts’ directory in the source code of the HDCytoData package. Here we have followed the standard setup for ExperimentHub packages – i.e. processed data objects that are ready to load into R, together with reproducible scripts saved in ‘inst/scripts’ – as described in the ExperimentHub vignettes. We believe this is a useful setup for these datasets. FlowRepository is primarily intended as a permanent public repository for .fcs files associated with peer-reviewed publications, which cannot be updated. FlowRepository is also primarily accessed via the web interface, so it would be much less user-friendly to only provide scripts that re-format the .fcs files after downloading. Providing these datasets as pre-formatted SummarizedExperiment and flowSet objects makes them much more easily accessible for users.

(2) In principle, any data types that can be formatted into SummarizedExperiment and flowSet formats could be added to the package. However, we believe it makes sense to keep the scope of the package relatively limited, to facilitate modularity and maintainability. For now, we plan to include only the current set of technologies, although in the future it may make sense to develop similar packages for other data types (e.g. imaging mass cytometry) (see Summary).

(3) According to the policies of FlowRepository, original .fcs files stored in FlowRepository cannot be updated after publication of the associated peer-reviewed paper. Similarly, data objects stored in ExperimentHub can only be updated manually by contacting the ExperimentHub maintainers. Therefore, we do not expect any major updates to the datasets currently stored in the HDCytoData package (except possibly minor bug fixes). We have included some additional text explaining this. We have also included a new vignette on “Contribution guidelines” (see comments for Reviewer 2).

(4) While users could indeed use the HDCytoData package to load these datasets in a consistent way for other purposes, we believe the main use cases for these particular datasets are for benchmarking and teaching / examples / tutorials. These datasets are well-characterized and have been studied in a number of previous publications, making them ideal for benchmarking. Formatting the datasets into consistent SummarizedExperiment and flowSet formats requires significant effort, so we expect this will mainly be worthwhile for datasets that can be re-used a number of times, e.g. for benchmarking.

(5-7) We have updated the text, main vignette, and help files to mention the size of the data files. The datasets range in size from 2.4 MB to 194.5 MB. We have also explained how to clear the local download cache, and updated the text to mention the expected level of experience with Bioconductor. We are not aware of a bulk download option in the ExperimentHub interface, so we have not included this. (If this functionality were added in the future, we believe it would better belong in the ExperimentHub package than in HDCytoData.)

(8) There is no simple way to convert between the SummarizedExperiment and flowSet formats. This is one of the major contributions of this package – we have pre-processed the datasets into both of these formats (with reproducible code saved in the ‘inst/scripts’ directory), so that users do not need to do this manually. We have included additional text to mention this.

(9) The additional columns of raw data (which are labeled as “none” in the “marker_class” column) contain additional information from the raw .fcs files from the mass cytometry machine, including barcodes for sample deconvolution, and event length and DNA content to identify live single cells. These columns are usually stored in the expression matrices in the original raw .fcs files, so we have also left them in the objects, e.g. for users who wish to check the pre-processing steps. We labeled these columns as “marker_class = none” to make them easier to identify, especially for users who are not already familiar with mass cytometry data. We have updated the help files to clarify that these columns are not needed for downstream analyses.

(10) Compensation for fluorescence spillover has already been performed by the original authors of the flow cytometry datasets, so users of these datasets do not need to perform this. However, users still need to apply a transformation (e.g. arcsinh), which we have described in the vignettes and help files.
